# Effects of Sound Interventions on the Mental Stress Response in Adults: Protocol for a Scoping Review

**DOI:** 10.2196/54030

**Published:** 2024-06-27

**Authors:** Marina Saskovets, Zilu Liang, Ian Piumarta, Irina Saponkova

**Affiliations:** 1 Faculty of Engineering Kyoto University of Advanced Science Kyoto Japan; 2 Department of Psychology St Petersburg University St Petersburg Russian Federation

**Keywords:** mental stress, anxiety, sound therapy, music therapy, voice-guided relaxation, voice-guided meditation, prosody, paralanguage, expressive sounds, psychoacoustics

## Abstract

**Background:**

Sound therapy methods have seen a surge in popularity, with a predominant focus on music among all types of sound stimulation. There is substantial evidence documenting the integrative impact of music therapy on psycho-emotional and physiological outcomes, rendering it beneficial for addressing stress-related conditions such as pain syndromes, depression, and anxiety. Despite these advancements, the therapeutic aspects of sound, as well as the mechanisms underlying its efficacy, remain incompletely understood. Existing research on music as a holistic cultural phenomenon often overlooks crucial aspects of sound therapy mechanisms, particularly those related to speech acoustics or the so-called “music of speech.”

**Objective:**

This study aims to provide an overview of empirical research on sound interventions to elucidate the mechanism underlying their positive effects. Specifically, we will focus on identifying therapeutic factors and mechanisms of change associated with sound interventions. Our analysis will compare the most prevalent types of sound interventions reported in clinical studies and experiments. Moreover, we will explore the therapeutic effects of sound beyond music, encompassing natural human speech and intermediate forms such as traditional poetry performances.

**Methods:**

This review adheres to the methodological guidance of the Joanna Briggs Institute and follows the PRISMA-ScR (Preferred Reporting Items for Systematic Reviews and Meta-Analyses Extension for Scoping Reviews) checklist for reporting review studies, which is adapted from the Arksey and O’Malley framework. Our search strategy encompasses PubMed, Web of Science, Scopus, and PsycINFO or EBSCOhost, covering literature from 1990 to the present. Among the different study types, randomized controlled trials, clinical trials, laboratory experiments, and field experiments were included.

**Results:**

Data collection began in October 2022. We found a total of 2027 items. Our initial search uncovered an asymmetry in the distribution of studies, with a larger number focused on music therapy compared with those exploring prosody in spoken interventions such as guided meditation or hypnosis. We extracted and selected papers using Rayyan software (Rayyan) and identified 41 eligible papers after title and abstract screening. The completion of the scoping review is anticipated by October 2024, with key steps comprising the analysis of findings by May 2024, drafting and revising the study by July 2024, and submitting the paper for publication in October 2024.

**Conclusions:**

In the next step, we will conduct a quality evaluation of the papers and then chart and group the therapeutic factors extracted from them. This process aims to unveil conceptual gaps in existing studies. Gray literature sources, such as Google Scholar, ClinicalTrials.gov, nonindexed conferences, and reference list searches of retrieved studies, will be added to our search strategy to increase the number of relevant papers that we cover.

**International Registered Report Identifier (IRRID):**

DERR1-10.2196/54030

## Introduction

### Background

The mental stress response exerts a profound influence at various levels in both the brain and the body. At the neural level, the amygdala (often referred to as “the emotional center”) plays a pivotal role by triggering rapid reactions to potential threats. The prefrontal cortex, responsible for rational decision-making, is also impacted. Additionally, the hypothalamus- pituitary-adrenal axis becomes activated, prompting the release of stress hormones such as cortisol, which affects bodily functions. Physiologically, the cardiovascular system responds by increasing heart rate and constricting blood vessels, redirecting blood flow to vital organs. These combined effects in the brain and body demonstrate the intricate interconnectedness of the stress response, underscoring its significance in shaping human behavior and health outcomes.

In the classical definition, stress is seen as a universal response to disrupted homeostasis [1]. From the point of view of the autonomic nervous system, we have 2 opposite reactions. One reaction is the stress response, which is accompanied by the arousal of the sympathetic nervous system, and a fight-or-flight response, which could lead to mydriasis, increased heart rate and force contraction, vasoconstriction, bronchodilation, glycogenolysis, gluconeogenesis, lipolysis, sweating, decreased motility of the digestive system, secretion of the epinephrine and cortisol from the adrenal medulla, and relaxation of the bladder wall. Opposing this reaction are the relaxation response, the activation of the parasympathetic nervous system, and the rest-and-digest response (which involves miosis, bronchoconstriction, increased activity of the digestive system, and contraction of the bladder walls) [2]. Chronic distress can worsen the quality of life, impair performance, lead to mental health problems, and aggravate bodily illnesses.

Ample evidence documented sound therapy’s integrative impact on the psycho-emotional and physiological outcomes, which makes it helpful for treating stress-related conditions such as pain syndromes, depression, and anxiety [3-6]. Sound therapy techniques have become widespread over the past decades, mainly focusing on music among other types of sound stimulation.

There is also evidence suggesting that a similar effect can be exerted by poetry therapy [7-9]. Similar features in the processing of speech and music are also evident at the level of brain activity. For example, in a study by Maess et al [10], it was shown that the Broca area, traditionally associated with language, is also involved in the processing of musical syntax. However, a comprehensive list of sound components that hold the potential for therapeutic influence remains ambiguously defined. There is a blind spot in understanding sound, such as speech therapeutic intervention and the impact of the spoken word.

In the case of speech, there are 8 paralinguistic features: pitch, tempo, loudness, resonance, timbre, intonation range, syllabic duration, and rhythm [11]. Despite this variety of features, studies on paralinguistic characteristics are scarce. The investigation of the effects of speech acoustics on changing mind-body conditions is just beginning. It is still unclear which combination of acoustic features (eg, prosody, pitch, loudness, and timbre) might work more effectively. We do not know whether there are individual differences in sound perception and, if so, whether they depend on cultural context, personal experience, characteristics of the listener’s nervous system, etc. We do not know whether a positive effect of sound is necessarily associated with subjective pleasure during listening, or whether there are conditions under which intervention would be beneficial regardless of subjective emotional preference. We plan to complete a scoping review of laboratory experiments, clinical trials, and randomized controlled trials to elucidate this field and investigate the capacity of sound stimulation to manage mental and physiological stress.

The main research question we intend to answer through this review is “what are the therapeutic factors of sound in the case of reducing mental stress and stress-related conditions in human adults?” For example, it might be rhythm, emotional prosody, environmental context of presentation of sound stimuli, individual preferences, or something else. Secondary research objectives will be to clarify body responses (physiological effects measured by biomedical technologies and devices) as well as subjective experiences (psychological effects measured by surveys and questionaries) associated with sound interventions.

We will incorporate investigations that delve into the responses of the hypothalamic-pituitary-adrenal axis and the autonomic nervous system as indicators of stress, supplemented by self-reported data and introspective surveys as markers of emotional stress. Our primary result of interest will be the neural mechanisms underpinning the therapeutic influence of sound. Beyond this, we are also interested in a comparison of delivery methods and sound sample choices for understanding the “active components” of sound in the therapeutic process.

### Novelty of This Work

There are reviews of music therapy and the effects of music, but there are currently no reviews that include a “polymodal” approach. This is particularly true of the therapeutic value of the human voice, the acoustic effects of which can be masked by the content of speech. The novelty of our review lies in a conceptually new perspective on sound interventions.

## Methods

### Overview

This section describes our protocol design, selection criteria, data extraction, and analytic methods.

### Protocol Design

In this study, we will follow the Joanna Briggs Institute Updated Methodological Guidance for the Conduct of Scoping Review [12]. It outlines the main steps to follow when conducting work using the evidence synthesis approach.

### Inclusion Criteria

To be selected for inclusion in our review, research articles must meet the following criteria.

#### Participants

Human adults are often exposed to diverse stress conditions that will be represented in a paper by the following keywords: stress-related abnormalities, anxiety, depression, stressful life conditions, personal crises, emergency, grief, loss, deprivation, burnout, occupational hazard, experimental stress, stress reduction, stress coping, relaxation skills, emotional flexibility, and mindfulness. These are associated with mental stress and can occur both inside the clinic and with healthy people, including artificial experimental stress.

This research excludes the outcomes within which health conditions can be treated with sound therapy but are not primarily caused by mental stress. However, they may be related to it because of difficult social conditions or disability. Meanwhile, the main malfunction can be found in a different area. For instance, this review does not consider the outcomes related to the pain syndromes or psychiatric disabilities (eg, bipolar disorder and schizophrenia). As for the other excluded outcomes, the work does not review Alzheimer or Parkinson disease, age-related cognitive changes, autism spectrum disorders, tinnitus, and hyperacusis.

Regarding excluded populations, our work does not take studies on animals, participants younger than 18 years of age, and participants with hearing disabilities into consideration.

#### Concept

The concept of the research is to study the sound intervention healing factors and provide an explanation of how and why a sound intervention works. All types of passive sound interventions, such as listening to instrumental music, poetry, human voices, nature sounds, etc, will be included. Our work will also clarify the conceptual basis that guides researchers in choosing one or the other sound sample for intervention, and why the authors consider some sound samples to be more effective than others.

As for kinds of sound intervention, various types of therapy (sound, music, poetry, and acoustic stimulation in hypnosis) are regarded, with sound intervention, guided relaxation, and guided meditation. Moreover, the study will consider issues with voice-guided imagery, affective prosody, expressive sounds, delivering emotions by music and voice, and voice-guided mental health.

#### Context

Regarding the context of the research, it incorporates the potential positive or harmful influence of sound in the areas of media, arts, and therapeutic and cultural practices. The study will remain in the psychophysiological context, which involves tracking instrumentally measurable body responses and subjective experiences. It will clarify the effectiveness of sound therapy as quantified by stress markers such as salivary cortisol, heart rate variability, electrodermal activity, neuroimaging markers, or other validated measures.

Considering the study types, the work will include not only randomized controlled trials and clinical trials but also laboratory and field experiments. We have deliberately limited the range of studies to controlled trials, clinical trials, and laboratory and field experiments to use more rigorous scientific information, as the field of sound therapy is often subject to a wide range of biases and speculations, which is currently beyond our research interest. All publications are in the English language. Although it is not recommended by the Joanna Briggs Institute Updated Methodological Guidance for the Conduct of Scoping Reviews to apply language restrictions, we do not currently have the resources for relevant translations from multiple languages. Even though automatic translators are available, they produce translations of different quality for different languages, which may misrepresent the results.

Considering the search strategy, we will use PubMed, Web of Science, Scopus, and PsycINFO or EBSCOhost databases. Since the neurobiology of music and sound therapy emerged as a separate field in the 1990s, the search strategy covers research from 1990 to the present day**.** We will also consider gray literature sources such as Google Scholar, ClinicalTrials.gov, nonindexed conferences, and searching the reference lists of retrieved studies.

### Evidence Screening, Selection, and Data Extraction

A clinical psychologist, MS, leads all aspects of the review, including literature search, extraction, screening, and data analysis. IS assists with the literature search and extraction and performs independent screening. Disagreements are resolved through discussion and consensus with other research team members, ZL and IP.

The complete string for the search strategy used across all identified medical databases (PubMed, Web of Science, Scopus, and PsycINFO or EBSCOhost) is formulated as follows: “(stress or anxiety or relax*) and (“sound therapy” or “music therapy” or “guided relaxation” or “guided meditation” or hypno* or ASMR or MBSR) and (prosody or song or poetry or voice or paralinguistics or paralanguage) NOT (children or infants or animal or teen).”

A 2-step process is used to select studies. First, we will screen citation titles, abstracts, and keywords and classify each citation as “include,” “exclude,” “unclear,” or “duplicate.” Next, full-text reports for “include” and “unclear” citations are read, with a final decision made about inclusion or exclusion. Reference management is performed in Rayyan. After the screening, we will construct a PRISMA (Preferred Reporting Items for Systematic Reviews and Meta-analyses extension) flow diagram [13] showing citations and full-text reports reviewed, included, and excluded. Data extraction, qualitative analysis, and data charting will then take place.

### Data Analysis and Synthesis

We will combine a descriptive analysis guided by the questions given at the beginning of this protocol and a thematic analysis that induces themes from the papers. Two reviewers will conduct independent data extraction from the full-text papers at this stage. Key data will be structured and placed in tables according to predetermined templates.

Two tables for primary data extraction will contain detailed information about each included study, as well as the relevant key findings regarding the review questions for original papers. The first table will serve as a summary of basic information and will include the sections mentioned in Textbox 1.

The second table will detail the “intervention” and “key findings” sections. We will elaborate 2 aspects of “intervention.” First, we will detail the parameters of the sound samples that the authors considered significant components of the intervention (pitch, tempo, rhythm, emotional tone, prosody, etc). Second, it is essential to provide details about the context of sound presentation, which can encompass a broad spectrum of factors, ranging from considering the individual features and preferences of the research participants to cultural context. For the “key findings” section, we are interested in detailing how mental stress was defined, and precisely which psychological and physiological markers were measured to track changes in the state of the participants. These may include autonomic responses such as heart rate variability or electrodermal activity, biochemical responses, brain activity measurements, behavioral responses, results from psychological questionnaires, and others.

After systematically describing the therapeutic aspects of sound in the presented papers, we will proceed with charting and categorizing. Based on the tables, we will review the effectiveness data of sound interventions, rank sound types according to their popularity of use, and identify gaps in research concerning potentially promising but infrequently used sound impact categories. We will also track which components of stress states (bodily responses, behavioral responses, subjective experiences, etc) are most sensitive to sound interventions. Furthermore, we will explore which varieties of clinical stress–related issues are most successfully addressed through sound therapy.

We will not conduct quality appraisal (risk of bias assessment) in this scoping review, which is consistent with the framework proposed by Arksey and O’Malley, as well as the Joanna Briggs Institute Methodological Guidance for Scoping Reviews [12].

**Sections for the summary table.**

**Sections**

- First author (year): indicating the first author and publication year.
- Sampling: the country where the study was conducted, sample size, and population type (nurses, students, patients from a specific clinic, etc).
- Study type: randomized controlled trial, clinical trial, laboratory experiment, etc.
- Intervention (sound type): the type of sound stimulation as described in the article (music, voice, nature sounds, etc).
- Target or conditions: the target condition or issue that the sound stimulation aims to impact (different mental stress types or stress-related clinical conditions such as anxiety, burnout, etc).
- Key findings: the primary findings of the study, such as clinical outcomes, body-mind responses, effectiveness, etc.

## Results

This section presents preliminary results that include searches of the main bases that do not consider the analysis of gray sources. According to the search strategy, we generated a search query that was used in 4 databases. Using the operator “and” we combined 3 fields. The first field consists of mental stress, stress related, or the opposite. The second field consists of interventions, in which sound plays a significant role. We included not only interventions based on sound explicitly but also those in which sound is not the central focus, such as speech instruction or performance. The third field consists of the terms related to the voice and the paralinguistic (intonation and acoustic) component. By combining the search fields in this way, we expect to identify a blind spot in the study of sound therapy, namely those papers that appeal to the therapeutic components of sound in natural speech, along the same lines as many music studies do.

The initial query was structured such that the “and” operator combined groups of factors, such as findings, intervention, and specification of studies related to acoustic characteristics of oral speech, and the “not” operator excluded an irrelevant portion of the population.

Running this query on PubMed generated 1467 results. Interestingly, including only “music therapy” in the “intervention” section generated 1370 results. However, when all other types of sound intervention except “music therapy” were included, only 191 results were returned. This illustrates a significant emphasis on research related to music and a lack of studies for other sound types. It is important to note that for therapies such as directed meditation, hypnosis, or other formal practices associated with oral speech, studies focusing on the conceptual and semantic aspects of the intervention are widely presented in these studies. Even so, they fall outside the scope of our review.

Within the focus of our work, it is crucial to highlight the paralinguistic component, which is invariably present in any oral speech interaction. For that purpose, we added a group of factors, related to the acoustic characteristics of voice, to our search query.

**Figure 1:**
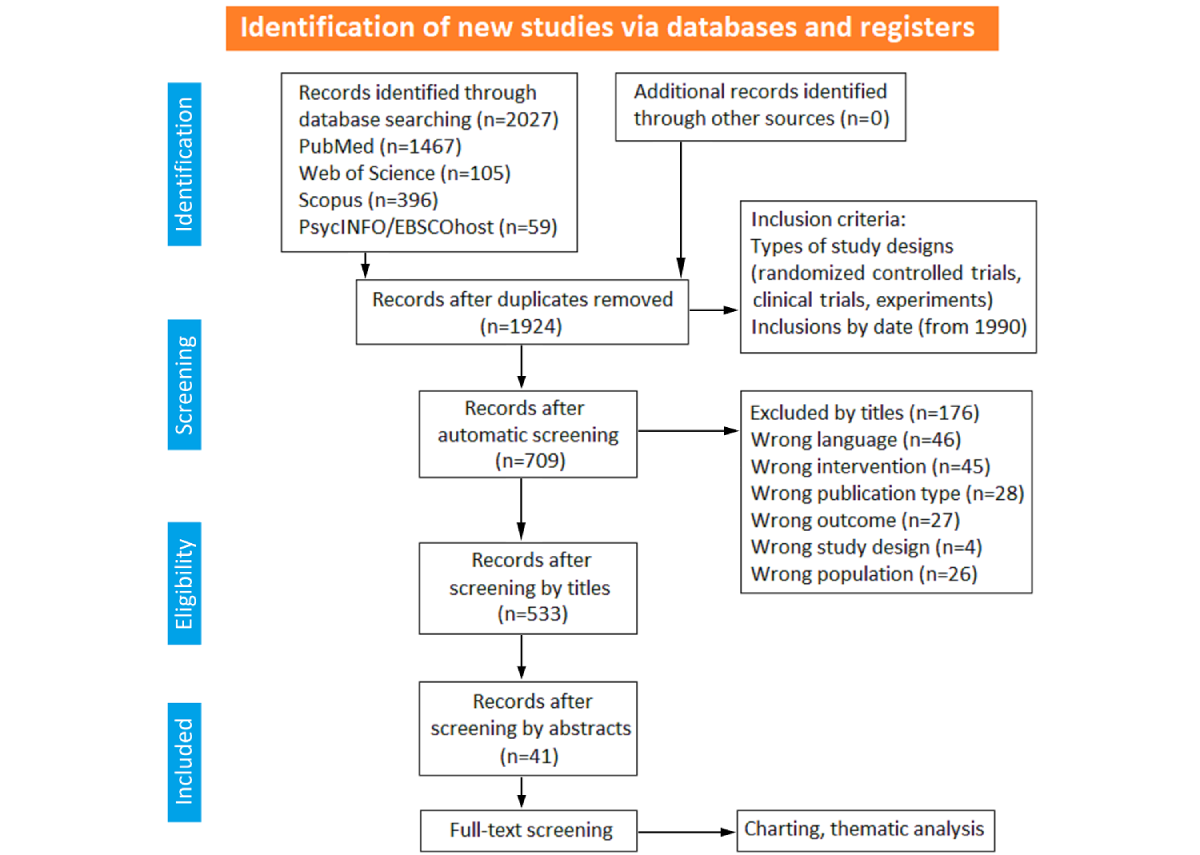
PRISMA (Preferred Reporting Items for Systematic Reviews and Meta-Analyses) flowchart.

Searching all the databases identified 2027 papers. After removing duplicates, 1924 papers remained. We performed an initial screening of those papers using Rayyan software. We first eliminated inappropriate papers by unambiguous, incontrovertible attributes such as year of publication, paper language, and study design. We then screened by title and abstract using the predefined legibility criteria outlined in the methodology section of this study. After abstract screening, we identified 41 eligible papers. As of October 2023, we are in the full-text analysis phase. Results have been organized according to the PRISMA flowchart format [13] (Figure 1).

## Discussion

### Principal Findings

The anticipated main finding of this study would be a comprehensive overview of empirical research on sound interventions, aiming to elucidate the mechanisms underlying their positive effects. The focus lies on identifying therapeutic factors and mechanisms of change associated with various sound interventions, including but not limited to music therapy.

**Figure 2:**
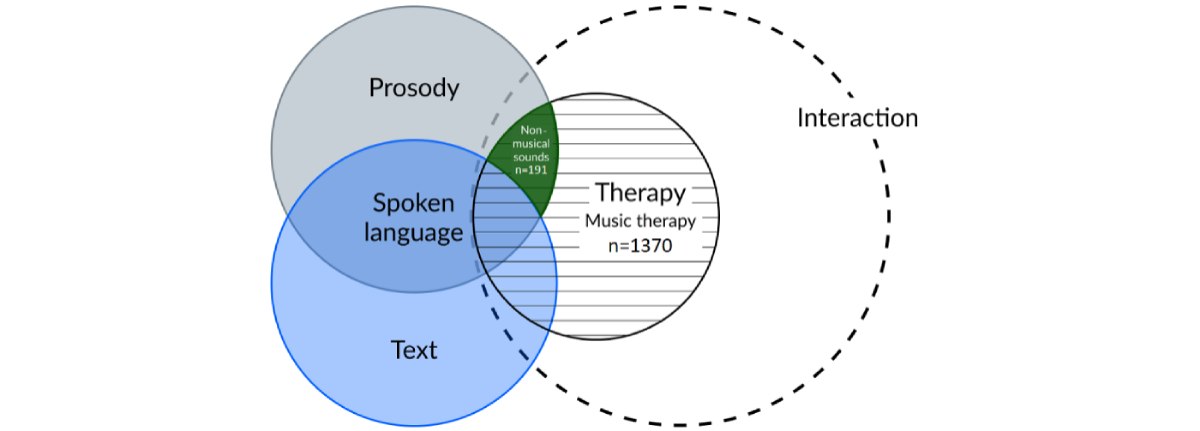
Field of study and research gap.

A preliminary search with various query options shows a lack of research beyond music therapies. In methods using natural speech, such as poetry therapy, guided imagination, and guided meditation, the acoustic component is masked by the semantic component (the part of the intervention that can be separated from the vocal expression and conveyed as text). We suggest grouping the therapeutic factors of sound as a potential way to overcome these blind spots (Figure 2).

Stimuli for sound intervention can be described at different levels of generality: starting with basic characteristics, such as frequency (pitch) and amplitude (volume), and then moving on to more complex processing levels, such as tempo, rhythm, and melody (the sound patterns that can be determined through analysis of spectro-acoustic characteristics) [11]. These parameters can be defined for any stimulus, whether it is music, speech, or a nature sound. We can then define the emotional-expressive component, in terms of basic emotions (calm, sad, joyful, ecstatic, etc), which remains a common characteristic for speech and music (modern programs for identifying emotions in voice and music use similar emotion classifications [14]) Thus, we see that dividing sound stimulation into the categories of “music”, “speech”, and “natural sounds” is a very high level of generalization that may hide some basic common features.

We noticed that many articles focus on higher-level attributes, for example, distinguishing groups of participants who listen to “nature sounds” versus “music,” without specifying which particular music and nature sounds were chosen. For example, the murmuring of a stream, the rumbling of a rockfall, the chirping of birds, and the screams of fighting cats can all be considered sounds of nature. These examples can provoke a wide range of emotional responses in a listener. Conversely, some sound examples are difficult to define unambiguously. An example might be a vocal expression in opera singing, modern electronic music, or traditional rituals and folklore performances. In some borderline cases, if the listener does not initially know the situational context, then it is difficult to determine whether a sound is speech, music, or from nature.

Following this observation, we consider situational, personal, and cultural contexts in our analysis. After a preliminary review of papers on abstracts, we found several papers focusing on factors such as personal preferences or freedom of selecting sound samples at the intervention time.

A cluster of papers also suggests country-centric interventions, such as traditional music or poetry, implicitly recognizing the importance of cultural context [15]. Often, both the authors and participants in these experiments hail from the country of origin of the music or poetry used in therapeutic sound interventions. However, only a few studies consider that the therapeutic effect may stem from the sound itself and cultural learning. In the latter case, such interventions may be more effective for native listeners. In our review, we aim to focus on these papers to explore the correlation of therapeutic factors of different levels.

### Limitations

Although the aim of the study is to elucidate the mechanisms underlying sound interventions, we may encounter an incomplete understanding of the mechanisms due to the lack of scientific consensus regarding strict definitions in this field. Considering its complexity and multifaceted nature, it may be more appropriate to conclude existing research approaches rather than the very mechanisms of sound therapy. Even a surface review at the level of abstracts shows us the fragmentation and ambiguity of the categorization used by the authors to compare different types of sounds. The range of characteristic analyses extends from the physical features (comparing sound effects of different frequencies), or grouping by context (live presence vs records and free choice of intervention vs predetermination), to categorization by abstract, culturally specific concepts (comparing the effectiveness of sounds by country of origin). Although the study mentions a comprehensive search strategy, it is possible that some trials were missed due to limitations in the search terms or databases used. In addition, this study focuses on the literature in English, which hides from us data published in other languages. This imposes some limitations on the understanding of global research on this topic.

### Conclusions

As an expected result of this study, we are going to identify different approaches and concepts underlying sound interventions. The comparison of different sound interventions could reveal specific strengths and weaknesses of each approach in addressing various psycho-emotional and physiological conditions. This could lead to more targeted recommendations for sound therapy apps. Moreover, by exploring the therapeutic effects of natural speech and poetry, the study might uncover additional benefits and potential apps of these sound forms beyond traditional music therapy. Also, we are going to categorize identified therapeutic factors based on their specific characteristics, such as acoustic properties, emotional associations, or cognitive engagement. The study might identify specific sound elements or combinations that are most effective for mental stress treatment leading to more targeted sound therapy protocols. Including gray literature sources could reveal valuable insights and potentially identify novel sound interventions or therapeutic apps not yet documented in mainstream research.

## Abbreviations

PRISMA: Preferred Reporting Items for Systematic Reviews and Meta-Analyses
PRISMA-ScR: Preferred Reporting Items for Systematic Reviews and Meta-Analyses Extension for Scoping Reviews

## Data Availability

Data sharing is not applicable to this article as no data sets were generated or analyzed during this study.
